# Splice-site identification for exon prediction using bidirectional LSTM-RNN approach

**DOI:** 10.1016/j.bbrep.2022.101285

**Published:** 2022-05-26

**Authors:** Noopur Singh, Ravindra Nath, Dev Bukhsh Singh

**Affiliations:** a Dr. A. P. J. Abdul Kalam Technical University, Lucknow, 226021, India; b Department of Computer Science, University Institute Engineering and Technology, Chhatrapati Sahu Ji Maharaj University, Kanpur, 208024, India; c Department of Biotechnology, Institute of Biosciences and Biotechnology, Chhatrapati Sahu Ji Maharaj University, Kanpur, 208024, India; d Department of Biotechnology, Siddharth University, Kapilvastu, Siddharth Nagar, 272202, India

**Keywords:** Splice-site, Intron, Exon, Machine learning, Deep learning, Bidirectional LSTM-RNN, DNA, Deoxyribonucleic Acid, RNA, Ribonucleic Acid, CDS, Coding Sequence, LSTM-RNN, Long Short-Term Memory Recurrent Neural Network, ANN, Artificial Neural Network

## Abstract

Machine learning methods played a major role in improving the accuracy of predictions and classification of DNA (Deoxyribonucleic Acid) and protein sequences. In eukaryotes, Splice-site identification and prediction is though not a straightforward job because of numerous false positives. To solve this problem, here, in this paper, we represent a bidirectional Long Short Term Memory (LSTM) Recurrent Neural Network (RNN) based deep learning model that has been developed to identify and predict the splice-sites for the prediction of exons from eukaryotic DNA sequences. During the splicing mechanism of the primary mRNA transcript, the introns, the non-coding region of the gene are spliced out and the exons, the coding region of the gene are joined. This bidirectional LSTM-RNN model uses the intron features that start with splice site donor (GT) and end with splice site acceptor (AG) in order of its length constraints. The model has been improved by increasing the number of epochs while training. This designed model achieved a maximum accuracy of 95.5%. This model is compatible with huge sequential data such as the complete genome.

## Highlights

• A deep learning-based method, bidirectional LSTM-RNN has been used for splice site identification for the prediction of exons.
• The built model has been trained and tested on genome data *Cryptosporidium parvum*.
• Model shows better accuracy over some other deep learning methods and is compatible with huge sequential data such as complete genome.
• The exons predicted by this method aid in protein modeling for specific drug targets.

## 1 Introduction

Recent research has shown that the machine learning approach serves to be a boon for different types of prediction, especially in the field of bioinformatics. In the last many years an increasing number of sequencing projects and the accessibility of entirely sequenced genomes create difficulty in finding gene sequences in an expeditious and decisive manner. In this research area, bioinformatics plays a major role. In reality, for the improvement of genome annotation, a number of bioinformatics tools and software have been developed that consider multiple and heterogeneous evidence sources. Genome annotation has two distinct phases: gene prediction and functional annotation. The process to identify the exact gene structure is described by the prediction phase, restricting the boundaries of exon and intron and the localization of genes on the genome. On the other hand, characterization of predicted genes, assigning them a biological function, and identifying their metabolic role or describing structural features are the criteria of functional annotation.

The whole genome sequence of an organism is actually a “blueprint”, that says that an organism's genome carries a set of instructions that recite its biological characteristics. The central dogma plays an indispensable role in unfolding the instructions by the process of transcription of the DNA into RNA and then RNA to protein. That means the DNAs are processed into messenger RNAs, which pass through the nuclear membrane and reach the cytosol, where they are translated to proteins. In the eukaryotic cell system, just after the transcription process, a process called “splicing” takes place. Splicing is the process in which the non-coding sequences of genes i.e., introns are removed and the coding sequences i.e., exons are spliced back which means joined so that the hnRNA (heteronuclear RNA) after splicing becomes mRNA (messenger RNA) and the translation process gets enabled.

Eukaryotes have a complex genome in which less than 5% of DNA carries protein-coding sequences and the rest are non-coding and untranslated regions of DNA. Identifying genes from DNA sequences is an important problem in bioinformatics. The region of the DNA sequence that codes for a protein are called the coding sequence (CDS). The main characteristic of a eukaryotic CDS is the organization of its structure into exons and introns. These exons and introns are separated by splice site regions. In general, there are four classes of exons: (i) 5′ exons, (ii) internal exons, (iii) 3′ exons and (iv) intronless exons [1]. Exons are the region of the gene that codes for protein to be expressed and introns are the non-coding region of the gene segment. The exon and intron sequences are characteristically envisaged as the sense strand of the double-stranded DNA (5′-3′). In general, in the coding sequence, the exon region starts with a start codon ATG and ends with one of the three stop codons, TAA, TAG and TGA. At least two exons are there in the coding sequence which makes us know that there is a minimum of one intron region and usually intron region starts with GT bases and ends with AG bases [2]. Splice sites are those sites that separate exons from introns and after splicing introns are removed and exons are joined. Introns are characterized by splice site donor (GT) and splice site acceptor (AG) [3]. These splice site donor and splice site acceptor acts as a signal in identifying splice sites in exon prediction methods.

The accuracy in the prediction of genes is indispensable for the accomplishment of computational gene discovery from genomes. Machine learning approaches and statistical techniques are being used for predictions that can reliably identify genes in anonymous sequences of DNA. Some of the machine learning approaches are; Hidden Markov Model, Artificial Neural Network, Deep Learning, etc.

Machine learning is a field of artificial intelligence that is growing continuously and contributing to its application in solving biological problems. In machine learning, the machine is made learned by improving the computer algorithms with experience [4,5]. In the field of bioinformatics, machine learning is performing so well such as in genomics and prediction of hidden factors of genomic sequences. Prediction of coding regions of DNA has always been a challenge for computational biologists due to the complex nature of the eukaryotic genome. As computational programming has become more comprehensive and evolving gradually, machine learning is playing an inevitable role in the field of bioinformatics. Deep learning is the subset of machine learning where neural network algorithms use a huge amount of data to learn [6]. The deep learning model can be comprehended as a unique type of artificial neural network that is designed in multiple layers.

The idea behind ANN is the human brain that is built up of multiple interconnecting neurons, i.e., nerves cells. In the context of artificial intelligence, the brain is a highly complex, nonlinear and parallel computer, whose structural constituents or basic units are “neurons” [7,8]. So, the machine can also perform tasks such as decision making, classification, and prediction as the human brain does [5]. Basically, in ANN, the processing elements, neurons are interconnected to each other in such a way that they are structured in three layers: the input layer, the hidden layer and the output layer. In advance of ANN, the hidden layer may be up to five. The information is sent by the input neurons, that build the input layer, to the hidden layer. The hidden layer neurons send the information to the output layer. The neurons of each of these layers contain some parameterized weights [8]. In machine learning, there are different types of neural network defined which falls under the category of deep learning. Deep learning is actually a subset of machine learning itself. The different types of neural networks like Artificial Neural Network (ANN), Convolutional Neural Networks (CNN), Recurrent Neural Networks (RNN), etc. play a great role in prediction programs.

## 2 Materials and methods

### 2.1 Long Short-Term Memory Recurrent Neural Networks (LSTM-RNN)

Long short-term memory-based Recurrent Neural Networks (LSTM-RNN) are basically a deep learning model. Deep learning or deep structured learning can be defined as a special kind of neural network composed of multiple layers. These networks are better than traditional feed-forward neural networks that need a fixed size input and give fixed-size output and are not designed for sequences or time-series data. A recurrent neural network (RNN) is designed for capturing information from sequences or time-series data. They can take variable size inputs; variable size outputs and they work well with these sequences (like DNA sequences) or time-series data. RNN is one such machine that has a combination of networks in the loop. The networks in the loop allow the information to persist. Each network in the loop takes input and information from the previous network and performs the specified operation in turn producing output along with passing the information to the next network [9]. In the case of multilayer RNN, the output calculated serves as the input of the next layer and thus helps in creating multilayer RNN, that is deeper the network better will be the accuracy. Some applications require only recent information while others may ask for more from the past. The common recurrent neural networks lag in learning as the gap between required previous information and the point of requirement increases to a large extent and in turn, it affects the accuracy of the model. But fortunately, Long Short Term Memory (LSTM) Networks, a special form of RNN are capable of learning such scenarios [10]. These networks are precisely designed to escape the long-term dependency issue of recurrent neural networks [11]. LSTM is actually the addition of little more interactions to RNN to increase the accuracy of the model.

Long short-term memory (LSTM) is an artificial recurrent neural network (RNN) based architecture used in the field of deep learning [11]. Unlike standard feed-forward neural networks, LSTM has feedback connections. It can not only process single data points (such as images), but also entire sequences of data (such as DNA/RNA). LSTM is a type of recurrent neural network (RNN) proposed by Hochreiter and Schmidhuber (1997) [12], [13].

**Fig. 1:**
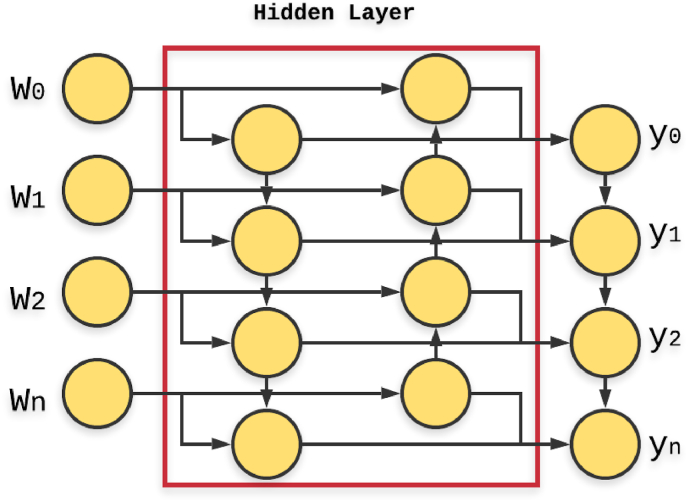
The architecture of Bidirectional LSTM in which both flow of backward and forward information is shown by the directed arrows in the hidden layer; w_0_, w_1_, w_2_ and w_n_ represent the input and y_0_, y_1_, y_2_ and y_n_ represent the output respectively.

In this paper, bidirectional LSTM (Long Short-Term Memory) RNN (Recurrent Neural Network) has been applied for the identification of splice-sites for the prediction of eukaryotic exons. Bidirectional LSTM structure permits the networks to preserve both backward and forward information from the two combined hidden states regarding the sequence data at each time step. In Fig. 1, both backward and forward information is clearly shown by the directed arrows in the hidden layer, w_n_ represents the input and y_n_ represents the output respectively. So that at any point in time, the information from both past and future are preserved using the hidden states [14,15]. This special feature of bidirectional LSTM increases the accuracy of the RNN model.

**Fig. 2:**
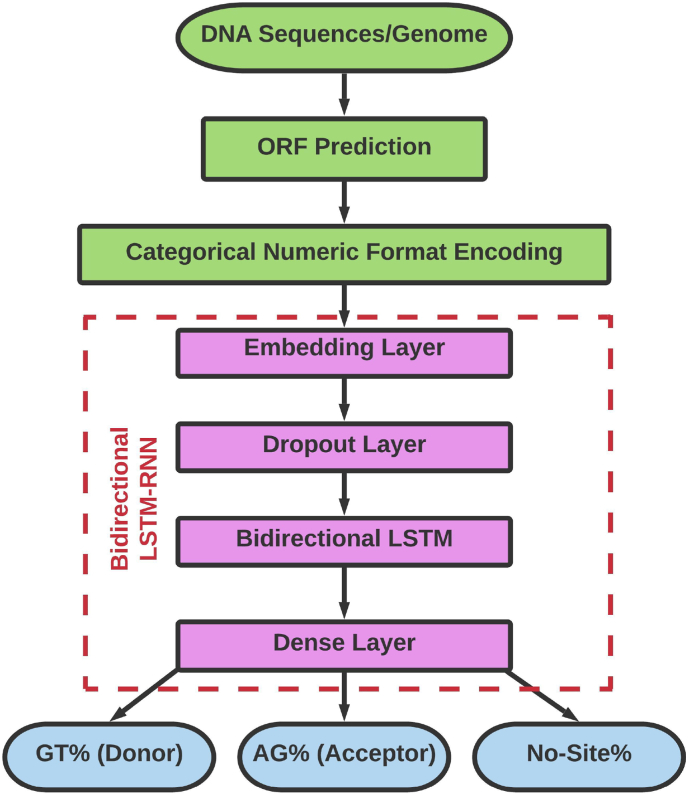
Proposed work plan using bidirectional LSTM-RNN model for splice site identification and prediction: Here in this figure, inputs for the model are the DNA sequences or may take complete genome. Then these DNA sequences are processed for ORF prediction; then these ORFs are then converted into categorical numeric format; Then these prepared datasets are passed through a Bidirectional LSTM-RNN model that consists of an embedding layer, a dropout layer, a bidirectional LSTM layer and a dense layer; after processing the output are donor, acceptor and no sites that is the identified and predicted regions.

The summarized work plan using the bidirectional LSTM-RNN model for the identification and prediction of splice sites has been illustrated in Fig. 2. Basically, inputs for the model are the DNA sequences or complete genome. Then, as a part of dataset preparation, open reading frames (ORFs) are predicted so that prediction progresses in a more precise manner. These ORFs are then converted into the categorical numeric format as deep learning models read input in numeric format. After dataset and model preparation the input dataset is passed through the bidirectional LSTM-RNN model. The splice site donor and acceptor regions i.e., GT and AG regions respectively are identified and predicted for the given DNA sequence. Details are described in materials and methods. On the basis of all theseinformation, exon predictions can be done more precisely.

LSTM networks are much suitable for classifications and predictions based on time series data, due to the lags of the unidentified period amid significant events in a time series. LSTMs were propagated to act on the vanishing gradient issue that can come across while training traditional RNNs [11]. An advantage of LSTM over RNNs and other methods is relative insensitivity to gap length in several applications. Introns always have two distinct nucleotides at either end. At the 5′ end the DNA nucleotides are GT [GU in the pre-messenger RNA (pre-mRNA)]; at the 3′ end they are AG. These nucleotides are part of the splicing site.

### 2.2 Data selection and training dataset preparation

Data selection is the foremost step for proceeding in prediction methods. The type of data here selected for the training dataset is basically a eukaryotic genomic DNA sequence in FASTA format. These nucleotide sequences are retrieved from the NCBI database (National Center for Biotechnology Information) and are stored together in a file.

Now next step is the preparation of the training dataset for the training of the model.

#### 2.2.1 Finding (open reading frame) ORF

The complete genome of *Cryptosporidium parvum (C. parvum)*, a protozoan, responsible to cause cryptosporidiosis that attacks the intestinal and respiratory epithelium of vertebrates has been selected as input for the bidirectional LSTM- RNN model for the identification and prediction of splice sites for exons. The complete genome of *C. parvum* comprises 8 chromosomes and a size of 9.1 Mb, comprising 3807 genes. The data used in this paper is from *C.parvum lowa II.* This organism has been selected as the complete annotation of its genome is available [16,17]. These annotations are set as benchmark data for the validation of the proposed model. The open reading frame (ORF) in the region of DNA sequence from the start codon (ATG) to stop codons (TAG/TGA/TAA) [18]. The ORFs are extracted from the complete genome of *C. parvum* by using ORFfinder [19] (https://www.ncbi.nlm.nih.gov/orffinder/). This program is executed for all the 8 chromosomes that are the complete genome of *C. parvum.* Minimal ORF length was kept at 150 and genetic code 4 was chosen as it includes the genetic code of protozoan because *C. parvum* belongs to the same group. Table 1 shows the chromosome-wise number of ORFs obtained. The total number of ORFs found from all the chromosomes was 785. All of these ORFs were stored in a single file.

**Table 1:** Result of ORFfinder for reference genome of *Cryptosporidium parvum* lowa II, median total length 9.1089 Mb and median GC% 30.2 (https://www.ncbi.nlm.nih.gov/genome/?term=cryptosporidium+parvum).

Chromosome no.	RefSeq (NCBI)	Size (Mb)	No. of ORFs
**1.**	NC_006980.1	0.88	87
**2.**	NC_006981.1	0.99	95
**3.**	NC_006982.1	1.1	93
**4.**	NC_006983.1	1.1	89
**5.**	NC_006984.1	1.08	100
**6.**	NC_006985.1	1.33	110
**7.**	NC_006986.1	1.28	117
**8.**	NC_006987.1	1.34	94

#### 2.2.2 Identifying splice site donor (GT) and acceptor (AG)

Here, along with the identification of the “splice site donor (GT) region” and “splice site acceptor (AG) region”, one more region, “No site region” was added to the program to increase the model's performance. A program has been developed to identify splice site donor, the GT region and splice site acceptor, the AG region. Algorithm 1 shows, that this program reads the ORFs as a string variable that finds the triplets that start with GT and stores the triplet's position in an array containing the positions of the GT region. In the same manner, the program searches for the AG region and stores its position in an array containing the positions of the AG region. After that, for the no-site region, the region that is not a potential splice site is considered a No-site region and its position has been also stored in an array containing a No-site region [20].

#### 2.2.3 Preparation of splice site classes

Now, the DNA sequences of the merged ORFs that were stored in a single file, were split into a sequence of window size 60 in such a manner that each sequence contains one of the splice-site.

Each of the classes contains such 37005 sequences with the window size 60 as the number of GT positions identified were 37005. The number of AG positions was also 37005 because the intron starts with GT and ends with AG, the numbers of GT and AG positions must be equal. The positions of No-site were also 37005 as it is the mean value of GT and AG positions. But all these predicted positions are not true donor splice sites (explained in the result and discussion section).

Three classes of splice site regions were prepared, donor (GT) splice site region, acceptor (AG) splice site region and no-site region. Each of the classes consists of 37005 sequences with a length of 60 nucleotides.

##### 2.2.3.1 Donor (GT) splice site region

For each position in the GT position list, 30 nucleotides were appended just before the GT position and 30 nucleotides just after the GT position. In this way, a nucleotide sequence of window size 60 containing the GT region in the middle is prepared. This class of training dataset has been labeled as 0.

##### 2.2.3.2 Acceptor (AG) splice site region

Similarly, for each position of AG in the AG position list, 30 nucleotides were appended just before the AG position and 30 nucleotides just after the AG position. In the same way, a nucleotide sequence of window size 60 containing the AG region in the middle is prepared. This class of training dataset has been labeled as 1.

##### 2.2.3.3 No-site region

Respectively, for each position of no-site in the no-site position list, 30 nucleotides were appended just before the no-site position and 30 nucleotides just after the no-site position. Likewise, a nucleotide sequence of window size 60 containing a no-site region in the middle is prepared. This class of training dataset has been labeled as 2. Algorithm 1 describes the execution details.

In machine learning, the series of sequences of all these classes prepared needs to be converted into numerical representations [20]. Traditionally, a technique called one-hot encoding is used to convert each of the nucleotide positions in the DNA sequence of a certain length into a 4-dimensional binary vector, which is most suited for Convolutional Neural Networks (CNN) [10]. As in this work, bidirectional LSTM-RNN has been used, this approach will be inefficient and hence categorical numeric format encoding has been used in which A=>1, G=>2, T=>3 and C=>4 are considered for the whole DNA sequence [21].

Algorithm 1 Training data set preparation

**Figure:**
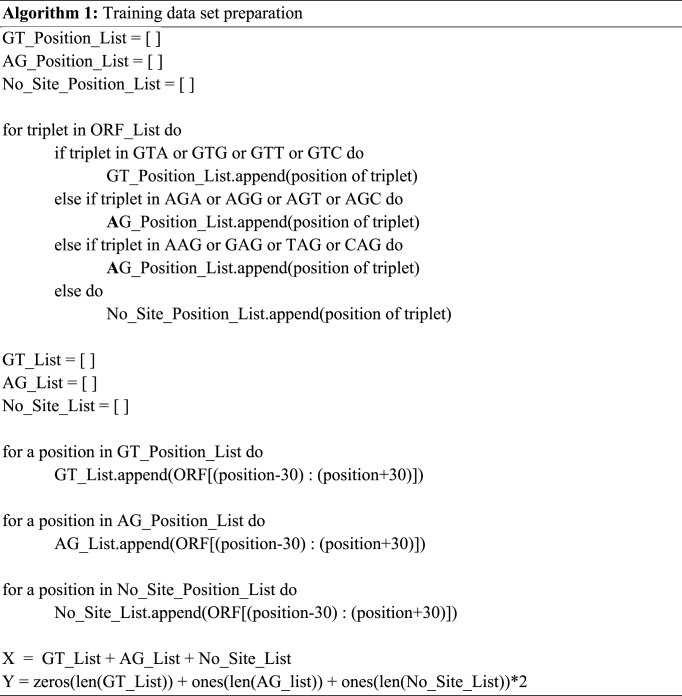

### 2.3 Designing bidirectional LSTM-RNN model

In this work, the bidirectional LSTM-RNN model has been designed using Keras of TensorFlow, a deep learning library and an API (Application Programming Interface) written in python that allows to define and train deep neural network models [22]. This model has been considered over traditional RNN and CNN models due to its good performance and ability to process huge sequential data with more accuracy and high processing speed. CNN models work for image processing and it does not work for sequence patterns [11]. The deep learning bidirectional LSTM-RNN model has been prepared by followings steps:

#### 2.3.1 Loading dataset

As mentioned in the preparation of splice site classes in the data preparation step (**2.2.3**), 37005 sequences were stored in each of the three classes respective to the positions of splice site regions. So, then a total of 111015 unique sequences were loaded in a NumPy array. Now, out of these 111015 sequences, 37005 sequences comprising of GT region were loaded GT sequence list, 37005 sequences comprising of AG region were loaded to the AG sequence list and 37005 sequences comprising the no-site region were loaded to the no-site sequence list. All the sequences of the GT sequence list were labeled to 0, the AG sequence list was labeled to 1 and the no-site sequence list was labeled to 2. The sequences of the above three lists were merged and the resulting list was stored to X (input). The labels of the above three lists were merged and the resulting list was stored to Y (output). The input and output resulting data list were split into 80–20 train-test datasets, which means, out of total data, 80% of data were stored as Train X (training input data) and Train Y (training output data) for the training of the model; rest of 20% data were stored as Test X (test input data) and Test Y (test output data) for testing of the model.

#### 2.3.2 Compilation of bidirectional LSTM-RNN model

**Fig. 3:**
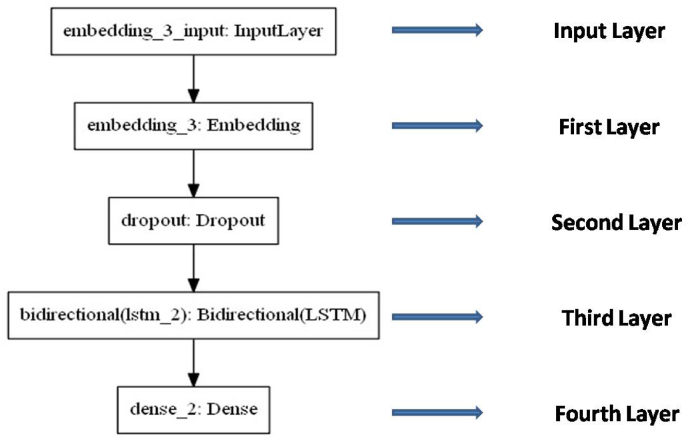
Bidirectional LSTM-RNN model representing each layer.

As in this work, four-layered LSTM-RNN sequential models are being used, the first embedding layer, which takes the input data in numeric format, with an input size of 60 and vocab size of 4 was added. Then, the second layer added is the dropout layer, which filters the results that are out of range [23]. After that, the third layer added is the bidirectional LSTM layer that has been introduced to RNN to improve the model performance, with 60 inputs. At last, the fourth layer added is the dense layer with 3 outputs and an activation softmax function that is the output function (Table 2 and Fig. 3). The result of the softmax function is interpreted as the probability distribution of the list classes. Once the model has been prepared, it gets compiled with probabilistic metrics i.e. loss = categorical_crossentropy class, adam optimizer and accuracy metrics to train the model [24].

**Table 2:**
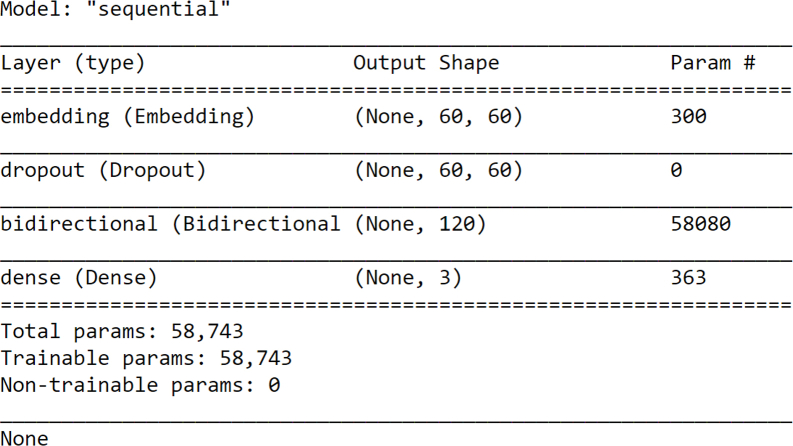
Summarized bidirectional LSTM-RNN Model.

## 3 Result and discussion

### 3.1 Training results for bidirectional LSTM-RNN model

As mentioned above, 80% of the total dataset prepared was used to train the model. That means the training dataset is Train X (training input data) and Train Y (training output data) which are comprised of 80% dataset. Table 3 shows the training data for both the X and Y vectors, where vector X represents all the nucleotide sequences with their window size and vector Y represents corresponding labels. For the training of bidirectional LSTM-RNN model, the method applied here is the Adam optimizer of Keras library that optimizes the multi-class loss function [25,26], categorical cross-entropy that calculates the loss between true labels and the predicted labels and accuracy metrics which computes that how often prediction equals labels.

**Table 3:** Training data comprising of 80% of total prepared dataset (80% of 111015).

Train_X	(88812, 60)
**Train_Y**	(88812, 3)

The compiled model was trained with input X, output Y and a set of 10 epochs 5 times (50 epochs).

**Fig. 4:**
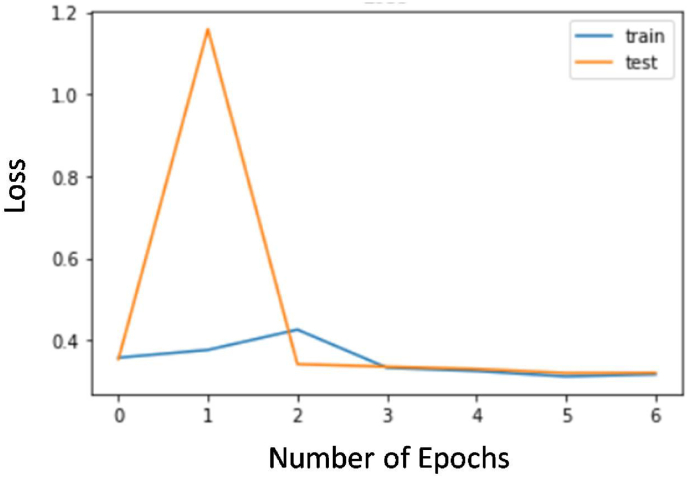
Loss curve of the model shows high training and test loss at beginning that gradually decreases and flattens thus proving a good fit model. As the curves of traing and test are very close, this is also a mark of a good fit model.

**Fig. 5:**
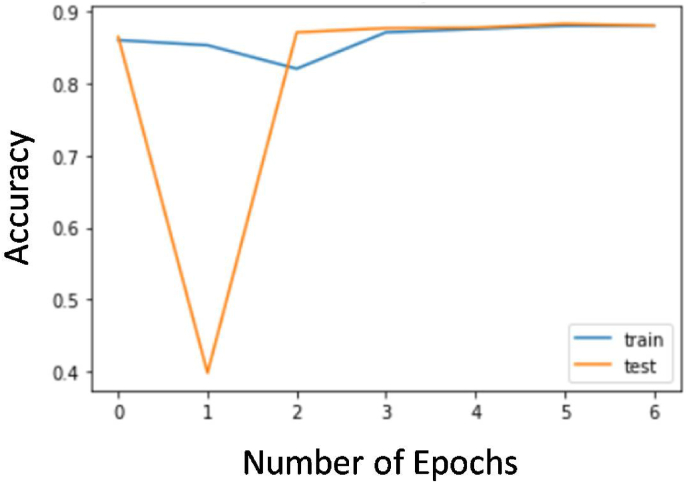
Accuracy curve of the model shows that the train and test curve are very close to each other thus proving a good fit model and pretty high accuracy.

The loss curves of the developed model for training and test data are shown in Fig. 4. The accuracy curves for the training and test model are shown in Fig. 5. This is apparent from the graph curves that as the number of epochs increases loss decreases and the accuracy of the model increases.

### 3.2 Testing of the bidirectional LSTM-RNN model

As described earlier in the methodology that 20% data of the total dataset that is Test X (validating input data) and Test Y (validating output data) comprises the 20% data of the dataset prepared were used to test the model. Actually, the model is evaluated with this 20% testing data. Table 4

**Table 4:** Testing data comprising 20% of total prepared dataset (20% of 111015).

Test_X	(22203,60)
**Test_Y**	(22203, 3)

**Fig. 6:**
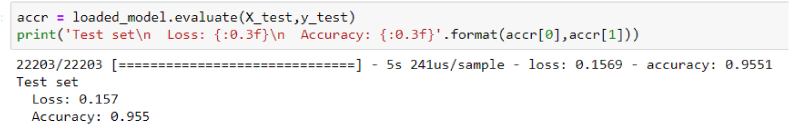
Test accuracy of the Bidirectional LSTM-RNN Model representing a test accuracy of 95.5% and loss 15.7%.

Shows the 20% test data with its X and Y vectors representing all the nucleotide sequences with their window size and corresponding labels respectively. The reason behind using this testing data is to test the abstraction ability of the trained model [27]. On evaluation, the model performance reaches the accuracy of 95.5% (Fig. 6) on the test data set after running 50 epochs, a set of 10 epochs 5 times.

After the evaluation of the model, a random whole genome sequence of *C. parvum* was taken as an input sequence to predict the exons from the trained and tested model. In the model, the length constraint for intron was added as a filter to improve the accuracy of the introns and exons prediction. A range of 70–100 intron length was set. After running the model, the prediction result comes in the form of labels 0, 1, and 2. This illustrates that in a particular window of sequences the counter shows a number of 0=> GT, 1 => AG and 2 =>no-site. The maximum number of counts for each label in one window will be the true prediction of the donor or acceptor sites for exon prediction. After applying the intron length constraint, 650 introns were predicted to possess an average length of 95. The number of predicted exons was 4325 with an average length of 1453. Table 5 shows the results of exon and intron prediction by the proposed model compared with the annotated genome of *C. parvum* [17]. This result shows that the model is validated with the annotated benchmark data of *C. parvum* with approximately 95% accuracy.

**Table 5:** Result of predicted exons and introns by the model with genome annotation.

	Predicted		Genome Annotation [17]	
	Exons	Introns	Exons	Introns
**Number**	4325	650	4553	688
**Average Length**	1453	95	1514	99

Table 6 represents the test accuracy of the proposed approach over other deep learning methods for splice site prediction. Here, the test accuracy of our proposed Bidirectional LSTM-RNN performance surpasses the other deep learning methods (Deep Belief Networks, Unidirectional LSTM and LSTM-RNN).

**Table 6:** Accuracy of proposed model and others.

	Deep Belief Networks [28]	Unidirectional LSTM [29]	LSTM-RNN [20]	Bidirectional LSTM-RNN (proposed approach)
**Accuracy**	0.888	0.820	0.943	**0.955**

As discussed earlier, bidirectional LSTM-RNN allows the neural networks to preserve backward as well as forward information from the hidden states of the sequence data so that the machine can learn much better. RNN models are designed in a way so that they can handle sequential data (like DNA sequence) well [30]. The reason behind the addition of LSTM to RNN was that only that LSTM solves the problem of ‘vanishing gradient’ and gives much better accuracy by adding extra interactions. Each state has some gradient, when we update each state and run our machine but then also the network cannot get better, this problem is called ‘vanishing gradient’ [31]. That is why Bidirectional LSTM-RNN has been used in this research work.

## 4 Conclusion

In this research paper, our proposed bidirectional LSTM-RNN approach for the identification and prediction of splice sites of eukaryotic DNA has been discussed. Bidirectional LSTM-RNNs are compatible with huge sequential data such as complete genome. The training speed of the model is increased in this approach. Results clearly show that only 50 epochs are sufficient to reach the accuracy level of 95.5%, this indicates the speed of the model. The loss curve clearly shows that the proposed model is a good fit model. Bidirectional LSTM-RNN gives the best results on the basis of accuracy. The accuracy of the model can be increased more by increasing the number of epochs or iterations. This model can be used efficiently in the prediction of more precise exons for various other eukaryotic genomes that will prove to be a great work and facilitate the study of comparative genomics. Also, the predicted exons can be extracted from the genome sequence by the exclusion of introns and concatenating the exons. These extracted exons can be used for protein modeling for specific drug targets.

## Ethics approval and consent to participate

Not applicable.

## Human and animal rights

No animals/humans were used for studies that are base of this research.

## Consent for publication

Not applicable.

## Funding

None.

## Declaration of competing interest

The authors declare no conflict of interest, financial or otherwise.

## Data Availability

The authors do not have permission to share data.
